# A Multicenter, Randomized, Controlled Trial of Osteopathic Manipulative Treatment on Preterms

**DOI:** 10.1371/journal.pone.0127370

**Published:** 2015-05-14

**Authors:** Francesco Cerritelli, Gianfranco Pizzolorusso, Cinzia Renzetti, Vincenzo Cozzolino, Marianna D’Orazio, Mariacristina Lupacchini, Benedetta Marinelli, Alessandro Accorsi, Chiara Lucci, Jenny Lancellotti, Silvia Ballabio, Carola Castelli, Daniela Molteni, Roberto Besana, Lucia Tubaldi, Francesco Paolo Perri, Paola Fusilli, Carmine D’Incecco, Gina Barlafante

**Affiliations:** 1 Clinical-based Human Research Department, Centre for Osteopathic Medicine—C.O.ME. Collaboration, Pescara, Italy; 2 Accademia Italiana Osteopatia Tradizionale, Pescara, Italy; 3 Neonatal Intensive Care Unit—Desio Public Hospital, Desio, Italy; 4 Neonatal Intensive Care Unit—Macerata Public Hospital, Macerata, Italy; 5 Neonatal Intensive Care Unit—Pescara Public Hospital, Pescara, Italy; Seattle Childrens Hospital, UNITED STATES

## Abstract

**Background:**

Despite some preliminary evidence, it is still largely unknown whether osteopathic manipulative treatment improves preterm clinical outcomes.

**Materials and Methods:**

The present multi-center randomized single blind parallel group clinical trial enrolled newborns who met the criteria for gestational age between 29 and 37 weeks, without any congenital complication from 3 different public neonatal intensive care units. Preterm infants were randomly assigned to usual prenatal care (control group) or osteopathic manipulative treatment (study group). The primary outcome was the mean difference in length of hospital stay between groups.

**Results:**

A total of 695 newborns were randomly assigned to either the study group (n= 352) or the control group (n=343). A statistical significant difference was observed between the two groups for the primary outcome (13.8 and 17.5 days for the study and control group respectively, p<0.001, effect size: 0.31). Multivariate analysis showed a reduction of the length of stay of 3.9 days (95% CI -5.5 to -2.3, p<0.001). Furthermore, there were significant reductions with treatment as compared to usual care in cost (difference between study and control group: 1,586.01€; 95% CI 1,087.18 to 6,277.28; p<0.001) but not in daily weight gain. There were no complications associated to the intervention.

**Conclusions:**

Osteopathic treatment reduced significantly the number of days of hospitalization and is cost-effective on a large cohort of preterm infants.

## Introduction

Preterm birth, defined as childbirth occurring at less than 37 completed weeks, is one of the major determinant of neonatal morbidities [1]. Long term effects are often associated with physical and psychological complications as well as higher economic costs [2]. Preterm birth rates have been reported to range from 7.5% to 12.5% of live births in developed countries [1,2]. These figures appear to be on the rise [3]. In Europe, the prevalence of preterm births account for the 6% (5.8 to 6.7) of all deliveries [2,4]. Preterm births can also be subdivided according to gestational age (GA): 5% of preterm births occur at less than 28 weeks’ (extreme prematurity), 15% at 28–31 weeks’ (severe prematurity), 20% at 32–34 weeks’(moderate prematurity), and 60–70% at 35–37 weeks’ (late prematurity). Moderate and late preterm births are five times more common than births before 32 weeks’ gestation, and currently, their public-health effects are understudied [5]. During the last three decades, there has been a 31% increase in the preterm birth rate in the USA, two-thirds of which were late preterm births (34–36 completed weeks’ gestation)[6]. Length of stay (LOS) is one of the major clinical outcomes used as proxy to explore effectiveness of interventions in the Neonatal Intensive Care Unit (NICU) setting. LOS seems to be associated with GA and birth weight [7]. Infants born at the earliest GA have the longest hospital stays as the highest risk to develop clinical morbidities. Moreover, it has been documented that preterm infants with lower birth weights increase the risk of severe medical complications and thus longer LOS [8]. In Italy, the national healthcare institute reported an average LOS per diagnostic related categories (DRG) ranging from four to 135 days [9]. In 2005, estimates indicated that the costs for preterm birth were more than US$ 26.2 billion (~€ 20 billion) and the average first year medical costs were US$ 32,325 (~€ 24,500) [10]. In Italy, the daily cost per infant varied between $264 (€200) and $660 (€500) in relation to infants’ health conditions [9], with preterm infants weighting significantly more on health care systems compared to term infants [11].

Complementary and alternative treatments (CAM) have been used in premature newborns. Osteopathy is a drug-free form of CAM, which uses a manual approach to diagnose and treat 'somatic dysfunctions'. Somatic dysfunctions are regarded as bodily areas which manifest an altered tissue texture, a restriction of range of motion, tenderness and asymmetry. Furthermore, these areas are characterized by a pro-inflammatory state as well as altered autonomic control. Although largely applied in the context of health care, particularly, in musculoskeletal problems, few osteopathic clinical trials have been conducted to investigate the role and the impact of osteopathic manipulative treatment (OMT) in the care of preterm infants. In 2011, Pizzolorusso and colleagues reported a significant decrease of the risk of LOS longer than 28 days (OR = 0.22; 95% CI 0.09 to 0.51) in newborns under OMT [12]. Two years later, Cerritelli and his co-workers demonstrated the positive effect of OMT compared to routine medical care in reducing days of hospitalization (-5.906; 95% CI -7.944 to -3.869) and costs [13]. Notwithstanding these positive findings, they should nonetheless be regarded as preliminary.

The aim of this multi-site nationwide randomized control trial was two-fold. Firstly, we investigated whether the results of previous studies would be replicated in a larger scale study. Specifically, we investigated the extent to which OMT is effective in reducing days of hospitalization in a sample of premature infants enrolled at three Italian NICUs. Secondly, we explored whether OMT is effective in reducing costs and daily weight gain.

## Materials and Methods

### Setting

From July 1st, 2012 to August 31st, 2013, we enrolled newborns admitted to three Italian secondary and tertiary NICUs, after the approval of the trial by the ethical committee of Macerata hospital (n°22/int./CEI/27239) and protocol registration (ClinicalTrials.gov number, NCT01645137). Parents or guardians provided written informed consent. The protocol for this trial and supporting CONSORT checklist are available as supporting information; see S1 Checklist and S1 Protocol.

### Patients

Preterm infants admitted to the NICU were eligible for enrollment. Infants born between 29 and 37 of gestational age, either gender, without congenital complications and with written informed consent signed were eligible for inclusion. The exclusion criteria, applied at both enrollment and during the study period included: lack of parental consent, the presence of any congenital or genetical disease, neoplasms, neurological, cardiovascular, urinary, hematological abnormalities, proven or suspected necrotized enterocolitis or abdominal obstruction, birth trauma, surgery patients, pneumoperitoneum, atelectasis, HIV, newborn from an HIV seropositive/drug-addicted mother and transferred to/from other hospital.

### Study type and outcomes

The primary objective of this multi-center randomized single blind parallel group clinical trial was to evaluate the effectiveness of OMT in LOS reduction. Secondary objectives were cost reduction and daily weight gain.

### Randomization and masking

We randomized patients from the first day of life using a 1:1 ratio to either the OMT group or the control group. Block randomization was performed according to a computer-generated randomization list using a block size of ten, and was stratified according to study center. The randomization was performed and stored in the coordinating center and an information technology consultant was responsible for the process. NICU staff were unaware of the study design and outcomes. Moreover, they were blinded to patients’ allocation, since all infants were touched by the osteopaths. Only osteopaths were aware of patients allocation. Moreover, the practitioners who performed OMT had no role in patient care decisions.

### Osteopathic procedure

Osteopathic procedures included a structural evaluation followed by a treatment. The structural evaluation was performed with the infant lying down in the open crib or incubator and was addressed to diagnose somatic dysfunctions [14]. It included rigorous and precise manual assessment of the skull, spine, pelvis, abdomen, upper and lower limbs to locate bodily areas with an alteration of TART (Tissue alteration, Asymmetry, Range of motion and Tenderness) criteria [14]. The treatment included the application of a selected range of manipulative techniques aimed at relieving the somatic dysfunctions. Techniques used were in line with the benchmarks on osteopathic treatment available in the medical literature and were limited to indirect techniques such as: myofascial release and balanced ligamentous/membranous tension. The whole session lasted 30 minutes, ten minutes for evaluation and 20 minutes for treatment. Registered osteopaths with experience in neonatology field performed OMT.

Infants allocated to the control group received only routine care and the structural evaluation. The structural evaluation lasted for ten minutes. To maintain blinding of the NICU personnel during the following 20 minutes, the osteopaths kept their position close to the incubator, with the hands inside but without touching the infant.

Newborns allocated to the study group underwent OMT plus usual care. We provided osteopathic care, for the entire period of hospitalization, twice a week, on Tuesdays and Fridays.

### Data entering and data export

We performed data collection using an ad-hoc locally developed software called EBOM-GCCN [13]. NICU staff collected daily nursing and medical records, from the time the infants entered the unit to the time of discharge.

Osteopaths collected osteopathic records twice a week when the osteopathic service was provided.

We also retrieved and included maternal data: pregnancy complications, single versus multiple gestation, fetal presentation, type of delivery, premature rupture of membranes, abruption of the placenta and any other complication.

Neonatal data collected included: gender, gestational age, infants small for GA, birth weight, neonatal complications (diagnosed at birth and during hospitalization), DRG at discharge.

An independent data and safety monitoring board periodically reviewed the efficacy and safety data. The group sequential method was used to characterize the rate at which the type I error was spent; the chosen spending function was the Lan—DeMets generalization of the O’Brien—Fleming boundary [15]. The monitoring board performed two interim analyses and was in charge of data export.

### Outcome measures

Primary outcome was the mean difference in days of hospitalization between study and control group. According to international guidelines, the following physiological conditions are required for discharge: generating heat at room temperature to maintain body temperature, coordinated sucking, swallowing and breathing while feeding; sustained pattern of weight gain; stability of cardiorespiratory function (no episodes of apnea/bradycardia for two to five days, free of supplemental oxygen support) [16]. There were no differences in formal discharge requirements at the three NICUs included.

Secondary outcome measures were:

- Daily weight gain, referred to as the net weight variation per day expressed in grams;
- NICU costs, calculated as NICU daily newborn expenses, according to local authorities; multiplied by the newborn’s LOS. Costs are estimated in Euros per day;
- Adverse events after OMT, i.e. perinatal deaths, oxygen desaturation, **bradycardia, tachycardia, apnea, bradypneas, tachypneas, constipation, diarrhea, inappetence, vomiting and any other neonatal morbidity and/or complication**.

### Statistical analysis

We reviewed relevant literature to determine the effect size. We assumed that the mean difference between study and control group was four days (SD 14), estimating an effect size of 0.3. We set the statistical power at 0.90 and an α-level equal to 0.01. This produced a sample size of 333 per group. To prevent loss of power, we increased the sample size up to 345 subjects per group. We conducted the primary analysis of the clinical trial according to per-protocol principle handling missing data using last observation carried forward (LOCF) imputation technique. We used arithmetic means and standard deviation as well as median, percentage and range to report the general characteristics of the study population. To compare the study group and control group at the baseline, we performed univariate statistical tests, student t test and chi square test. To study the independent effect of OMT on primary and secondary endpoints, we applied a linear regression model. To indicate statistical difference, we considered two-tailed P values of less than 0.01. To describe imbalances between groups, we used mean or RR with 95% CI. We computed effect sizes stratifying by gestational age. We utilized R statistical program for data analysis[17].

### Cost analysis

We performed ordinary least squares regression to investigate the average hospitalization costs among infants after adjusting for gender, gestational age, LOS, birth weight, OMT, time to first OMT, DRG and NICU centers. Cost data were extracted from 2012–2013 administrative databases of the Regional Office of the Ministry of Health of Marche, Abruzzo and Lombardia where the NICUs of the present RCT are located. To compute the precise cost, we used the reimbursement allocated to each DRG from the Istituto Superiore di Sanità (National Healthcare Institute). Specifically DRGs and reimbursements considered were 386 (12.932.69€), 387 (7.450.09€) and 388 (3.757.22€)[9]. As for the study group, OMT costs took into account fees from health insurance companies and the cost for each treatment was theoretically set at 20.00€ (FASDAC 2012) [18]. Cost estimates were adjusted for inflation to 2013 euros using the Medical Component of the Consumer Price Index.

## Results

**Fig 1:**
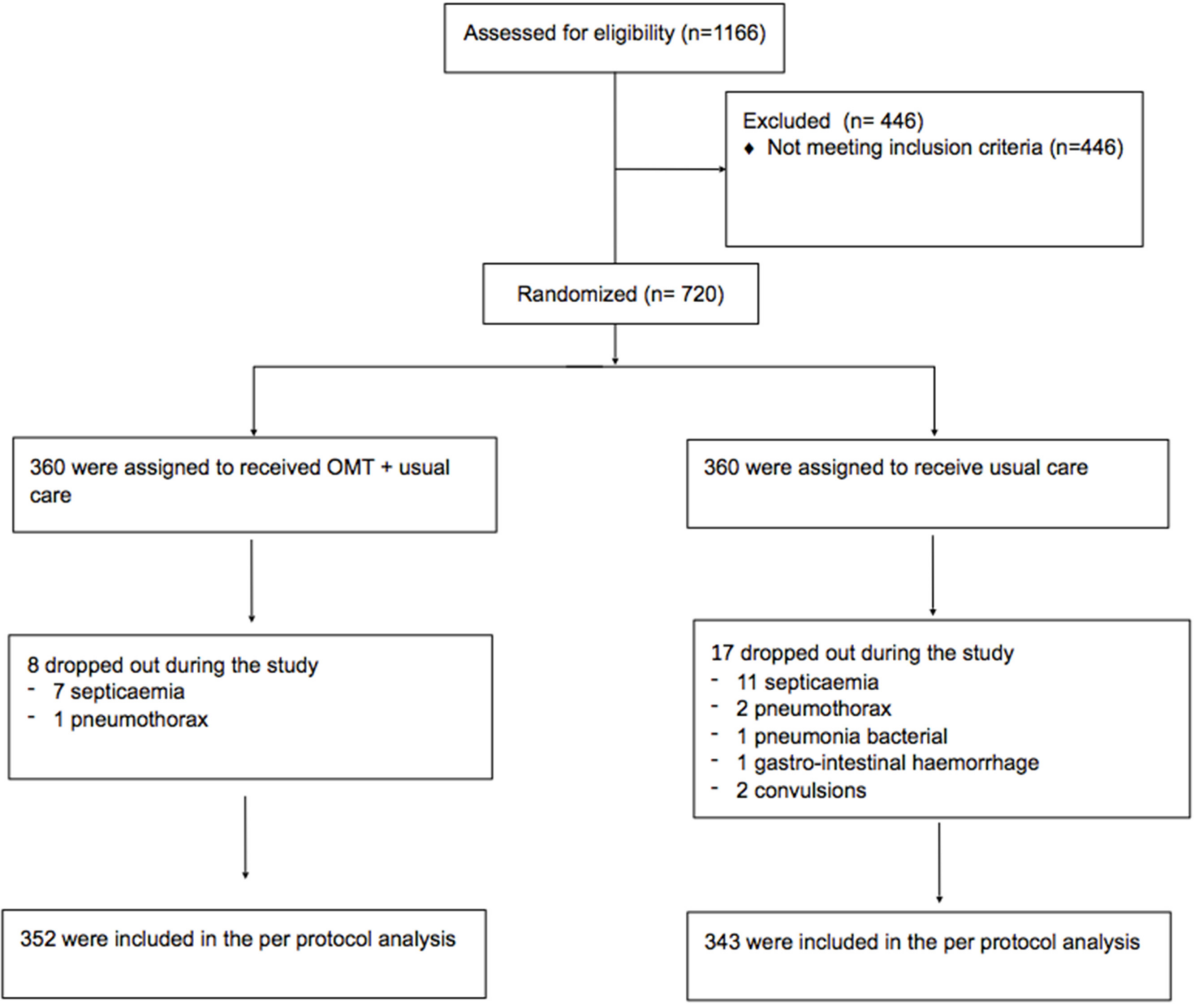
Flow chart of the study selection.

1169 newborns were assessed for eligibility, of whom 695 were enrolled and randomized in study group (n = 352) and control group (n = 343) (Fig 1). No significant imbalances were showed at the baseline in terms of demographic and clinical variables (Table 1). The only variable found statistically significant was other conditions (ICD-9 codes 760.8, 760.9, 761.8, 761.9) related to mother’s pregnancy.

**Table 1:** Description of the study population at the enrollment.

	Study group (n = 352)	Control group (n = 343)	p>ItI
**Neonatal**			
**Males** *	182 (51.7)	178 (51.9)	1
**Patient age (days of life)**	3.7 (2.3)	3.6 (2.2)	0.99
**Gestational age (w)**	34.3 (2.3)	34.4 (2.2)	0.44
**GA stratification**			0.64
***29–31***	50 (14.2)	49 (14.3)	
***32–34***	117 (33.2)	103 (30.0)	
***35–37***	185 (52.6)	191 (55.7)	
**Birth weight (gr.)**	2274 (748.9)	2325 (713.4)	0.36
**Small for gestational age** *	23	23	1
**Complications** §			
*** Jaundice*** *	78	73	0.78
*** Esophageal reflux*** *	1	2	0.54
*** RDS*** *	43	42	1.00
*** Endocrine & metabolic*** *	44	53	0.26
*** Other conditions*** *	44	43	1.00
**DRG** *			
*** 386***	21 (6.0)	70 (20.4)	
*** 387***	140 (39.8)	73 (21.3)	
*** 388***	191 (54.2)	200 (58.3)	
**Maternal**			
**Total number of women**	332	332	
**Single gestation** *	312	320	0.57
**Multiple gestation** *	20	12	0.17
**Vaginal delivery** *	288	275	0.68
**C section** *	44	57	0.23
**Cephalic presentation** *	348	337	0.5
**Breech presentation** *	4	6	0.5
**Pregnancy** §§			
***No complications*** *	294	302	0.12
***Gestational diabetes*** *	9	9	1.00
***Infections*** *	5	8	0.31
***Other conditions*** *	22	11	0.05
***Placenta abruptio*** **	1	1	1.00
***PROM*** **	1	0	0.31
***Hypertension*** **	0	1	0.31

RDS: respiratory distress syndrome. DRG: diagnosis related groups at discharge. PROM: premature rupture of membrane. Numbers are mean(sd). P value from t-test.

*n(%), p value from chi-square test.

^§^complications were classified according to ICD-9 codes.

^§§^pregnancy data were classified according to ICD-9 diagnosis codes.

**n(%), p value from Fisher exact test.

The study group received a median of 2 OMT sessions (range 1–17) and the time to the first OMT was 3.7 days after birth (SD 2.3) [median (range): 3 (0–12)].

### Primary outcome

LOS was used as primary outcome for estimating the effectiveness of OMT compared to usual care only. The average hospitalization for the study group and control group was 13.8 (8.1) and 17.5 (14.5) respectively (p value <0.001). Multivariate analysis showed that preterm infants allocated to the study group reduced LOS of almost 4 days (-3.944; 95% CI -5.548; -2.341; p<0.001; effect size = 0.31)(Table 2). GA was found to be associated with a reduction of LOS (-1.581; 95% CI -2.091; -1.070; p<0.001) as well as weight at birth (-0.001; 95% CI -0.003; -0.0003; p = 0.02).

**Table 2:** Results of multivariate linear regression for length of stay and weight gain.

	LOS			Weight gain		
	Estimate	95%C.I.	p>|t|	Estimate	95%C.I.	p>|t|
**Gender**	-0.277	-1.887; 1.332	0.73	-1.354	-6.400; 3.690	0.6
**Gestational age (w)**	-1.581	-2.091; -1.070	<0.001	-0.642	-2.252; 0.967	0.43
**Birth weight (gr)**	-0.001	-0.003; -0.0003	0.02	-0.007	-0.012; -0.002	<0.01
**OMT**	-3.944	-5.548; -2.341	<0.001	2.413	-2.612; 7.437	0.35

LOS = length of stay; OMT = osteopathic manipulative treatment.

### Secondary outcomes

#### Costs

The mean cost per newborn in the study group was 6,277.28 € and 7,863.29€ for the controls. This led to a difference between the two groups of 1,586.01€ (95% CI 1,087.18 to 6,277.28; p<0.001) per newborn. Considering the entire study sample, the use of OMT saved on average 550,348€ (95% CI 360,551 to 740,410). Ordinary least square analysis showed that OMT reduced hospital costs by 1,250.65 € per newborn per LOS (95% CI -5.548 to -2.341; p<0.001) (Table 3).

**Table 3:** Results of ordinary least square regression for cost estimates.

	Costs (2013€)		
	Estimate	95%C.I.	p>|t|
**Gender**	-12.87	-447.25; 421.51	0.95
**Gestational age**	-187.93	-329.39; -46.47	<0.01
**Birth weight (gr)**	-0.41	-0.84; 0.02	0.06
**LOS**	113.99	93.69; 134.30	<0.001
**OMT**	-1250.65	-1690.72; -810.59	<0.001

LOS = length of stay; OMT = osteopathic manipulative treatment.

#### Weight gain

No statistically significant associations were found between OMT, gender, gestational age and the average weight in grams per kilo per day. The only factor associated to weight gain was weight at birth (-0.007; 95% CI -0.012 to -0.002; <0.01) (Table 2).

#### Subgroup analysis

**Fig 2:**
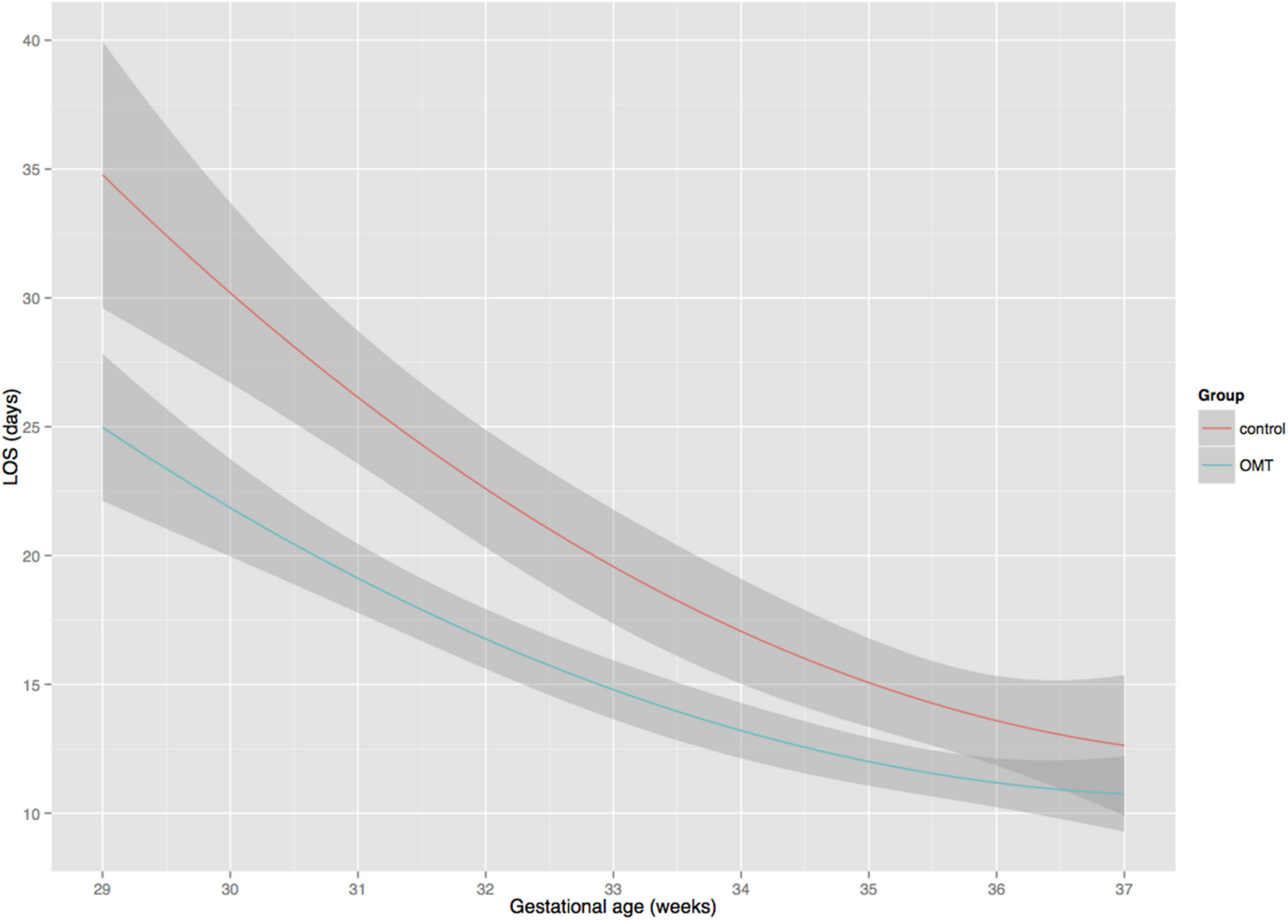
Relationship between LOS and GA by study group. Mean total and 95% CI length of hospital stay (LOS) measured in days per gestational age (GA) measured in weeks. Groups are: control (red) and OMT (cyan). A linear quadratic effect of LOS on GA is showed in both groups but the use of OMT significantly reduced LOS along different GAs (up to 36 weeks) compared to control group.

Subgroup analysis by gestational age was conducted to investigate any specific effect associated with the use of OMT. Considering gestational age as a continuous variable, Fig 2 showed the linear quadratic effect of GA on LOS (OMT group: y = 282.34–14.44x + 0.19x^2^; control group: y = 392.47–19.83x + 0.26x^2^). The use of OMT significantly reduced LOS along different GAs. The higher the prematurity the more effective the use of OMT on LOS. Furthermore, categorizing GA according to sub-categories of preterm birth, the study showed that OMT had a large effect on younger preterm infants (effect size (ES) = 0.68), medium effect on moderate preterms (ES = 0.40) and small effect on late preterm infants (ES = 0.22).

#### Adverse events

There were no perinatal deaths in either group. The survival rate at discharge was 100% in both groups. A drop-out analysis showed that the rate in the study group was 2.2% (8/360) compared to 4.7% (17/360) in the controls (X^2^ = 3.36; RR = 0.47; 95% CI 0.21–1.08; z = 1.76, p = 0.07). Moreover, no morbidities and/or complications were observed after the application of OMT.

## Discussion

This prospective, randomized, multi-center trial showed that OMT is effective in reducing LOS in a population of preterm infants. The effect size was modest and the number of days was reduced by 4. The use of OMT produced a net saving of almost 1,600€ per newborn, determining an overall net saving of more than 500,000€ during the 14 month study period. Consistent with results from previous single-NICU studies[12,13,19], the findings from the present trial confirmed the clinical effectiveness of OMT in the treatment of preterm infants. In 2013, Cerritelli et al showed larger benefit of OMT on LOS reporting a reduction of almost 6 days [13] while Pizzolorusso et al demonstrated a smaller decrease of LOS although significant [19]. These differences are essentially secondary to the populations studied. The former included both low-birth-weight infants as well as moderate and late preterm infants while the latter enrolled moderate and late preterm newborns only.

Length of stay is considered one of the major factor that contributes to the cost of hospitalization. Considering the cost-benefit of the present trial, findings from previous studies are also confirmed. Cerritelli et al.'s and Pizzolorusso et al.'s studies showed cost reductions in line with findings obtained in this multi-RCT [13,19]. Further analysis demonstrated that newborns born in Pescara’s hospital have a longer LOS. This could be explained by the different type of NICU level.

As far as adverse events were concerned, none were recorded during the study period. This result additionally confirms foregoing trials [12,13,19]. This study was six times as large as the largest, previous similar studies [13,19]. Moreover, it included three different NICU sites. Additionally, the use of a robust outcome measure, length of stay between entry and discharge, was more likely to be closely related to clinical outcomes.

Limitations were in terms of sample selected, mainly preterm clinically stable, and NICU costs derived form a standardized reimbursement form. Another concern was the few number of protocol variations, including missing data for additionally secondary outcomes calculation. Furthermore, mother’s pregnancy data was not systematically collected during the entire period of gestation but obtained at delivery. Although it could be argued that the day of discharge is influenced by the day of the week as well as from the neonatologist and NICU [10], randomization, concealment and precise discharge parameters established could eventually have decreased the risk of bias.

Several biological speculations could be addressed to explain the present results. Preterms have been demonstrated to have higher levels of pro-inflammatory substances [20] and a sustained increase of autonomic tone [21]. OMT has been shown to produce a parasympathetic effect [22] as well as anti-inflammatory action [23]. Although OMT findings were reported on different study samples, a 'neuro-biological' hypothesis could be theorized. Osteopathic manipulations could reduce the release of cytokines and the sympathetic activity creating a cascade of biological and neurological events, currently understudied in newborns, that modulate the inflammatory and autonomic nervous system mechanisms.

As far as possible impacts on health care system are concerned, the present study could be considered a successful example of integrated medicine. Since decades, the WHO has been encouraging multidisciplinary collaborations to enhance quality of practice. This led to include some traditional, complementary and alternative medicines within health care services [24]. In the context of NICU, team working has been tested since mid 90s [25] to implement procedures and deliver better practices [26]. However, to date, a fully integration of multidisciplinary collaborations is still limited [27], although promising results in terms of clinical effectiveness and reduction of costs [28–31]. **Therefore, despite the international political and research agenda that formally support a successful integration of different medical fields, lack of local funding and/or political will prevent further and more robust collaborations. The present study could open a discussion table of value to government policy-makers, regulators, researchers and health-care practitioners to debate on better evidence-based multidisciplinary practices**.

## Conclusion

As in the previous, smaller single NICU trials, the results of the present multi-center study provide further compelling evidence that among infants born prematurely, the osteopathic manipulative treatment reduces days of hospitalization and costs. Together with the prior results[12,13] and unpublished data that show the effectiveness of osteopathic manipulative treatment in preterm infants, the current findings suggest that health care guidelines for hospitalized infants should be revised to encourage the use of complementary evidence based interventions—specifically osteopathy.

Further studies exploring the effectiveness of osteopathic manipulative treatment among disease-specific preterm infants groups are required. Additionally, further prospective long-term follow-up to middle age is warranted to establish whether preterm infants osteopathically treated can change the risk of neurodevelopment sequelae, metabolic disorders and other co-morbidities related to their small size at birth and subsequent rapid catch-up in growth.

## Supporting Information

S1 Checklist CONSORT checklist. (DOC)

S1 Protocol Trial protocol. Description of the research protocol used for the current trial.
(DOC)
