# Supplementary material for: A Multicenter, Randomized, Controlled Trial of Osteopathic Manipulative Treatment on Preterms
Source: PLoS One. 2015 May 14;10(5):e0127370. doi: 10.1371/journal.pone.0127370 (PMC4431716; doi:10.1371/journal.pone.0127370)

EFFECTIVENESS OF OSTEOPATHIC MANIPULATIVE TREATMENT IN NEONATAL INTENSIVE CARE UNITS: PROTOCOL FOR A MULTICENTRE RANDOMIZED CLINICAL TRIAL

Cerritelli F^1,2^, Pizzolorusso G ^1,2^, Renzetti C ^2^, D’Incecco C^3^, Fusilli P^3^, Perri FP^4^, Tubaldi L^4^, Barlafante G ^1,2^

1 EBOM – European Institute for Evidence Based Osteopathic Medicine, Chieti, Italy

2 AIOT – Accademia Italiana Osteopatia Tradizionale, Pescara, Italy.

3 Neonatal Intensive Care Unit - “Spirito Santo” Civil Hospital, Pescara, Italy.

4 Neonatal Intensive Care Unit - Macerata Public Hospital

ABSTRACT

Introduction

Neonatal care has been considered one of the first priorities for improving quality of life in children. In 2010 the 10% of babies were born prematurely influencing national health care policies, economic action plans and political decisions. The use of complementary medicine has been applied to the care of newborns. One previous study documented the positive effect of osteopathic manipulative treatment (OMT) in reducing newborns’ length of stay (LOS). Aim of this multicentre randomized controlled trial is to examine the association between OMT and LOS across three NICUs.

Methods and analysis

690 preterm infants will be recruited from three secondary and tertiary NICUs from north and central Italy and allocated into two groups, using permuted-block randomization.

The two groups will receive standard medical care and OMT will be applied, twice a week, to the experimental group only. Outcome assessors will be blinded of study design and group allocation. The primary outcome is the mean difference in days between discharge and entry. Secondary outcomes are difference in daily weight gain, number of episodes of vomit, regurgitation, stooling, use of enema, time to full enteral feeding and NICU costs. Statistical analyses will take into account the intention-to-treat method. Missing data will be handled using last observation carried forward imputation technique.

Ethics and dissemination

Written informed consent will be obtained from parents or legal guardians at study enrollment. The trial has been approved by the ethical committee of Macerata hospital (n°22/int./CEI/27239) and it is under review by the other regional ethics committees. Results from this trial will be disseminated through scientific medical journals and conferences.

Trial registration

This trial has been registered at [www.clinicaltrials.org](http://www.clinicaltrials.org/) (identifier NCT01645137).

INTRODUCTION

Neonatal care has been one of the major focuses of the global health system policies, in terms of services delivered, to reduce neonatal mortality and morbidity. The last report of the WHO showed that more than 1 in 10 infants are born prematurely, resulting in 15 million premature infants worldwide in 2010 ^1^. In spite of expensive neonatal intensive care units (NICUs), structural changes in the health care system have led to evidence-based guidelines that reduce preterm infants hospitalization and deaths. A large rate of US pediatric hospital stays is secondary to neonatal conditions that rank among the most expensive items in the list of services provided for children ^2^. The highest average cost per infant is for preterm newborns with gestational age (GA) between 24-31 weeks, followed by those between 32-36 weeks, as opposed to the general population ^3^. Costs per surviving infant generally decrease with increasing GA. In the United States, preterm/LBW infants account for half the hospitalization costs of all newborns and one quarter of overall pediatric costs ^4^. In Italy, the cost per infant per day ranged between €200 and €500 according to infants health conditions ^5^.

Length of stay (LOS) in NICUs is strongly associated with GA and birth weight ^6^. Infants delivered at the earliest GA have the longest hospital stays, partly because of the higher incidence of medical complications in very low birth weight (VLBW) infants. The italian healthcare institute reported an average LOS per different diagnostic categories ranging from 4 to 34 days ^5^.

However, compared to term infants, premature infants are unique in their need to attain not only medical stability but also physiologic maturity, including adequate temperature control, cessation of apnea and bradycardia, and adequate feeding behavior, before they are safely discharged to home ^7,8^.

Patterns of hospitalization of preterm infants are also associated with the presence of clinical symptoms of abnormal gastrointestinal function such as vomit, regurgitation, gastric residuals and functional constipation ^9-11^.

Osteopathy is a form of drug-free non-invasive manual medicine, designated as complementary and alternative medicine (CAM). It relies on manual contact for diagnosis and treatment ^12^. It respects the relationship of body, mind and spirit in health and disease; it lays emphasis on the structural and functional integrity of the body and the body's intrinsic tendency for self-healing. Osteopathic practitioners use a wide variety of therapeutic manual techniques to improve physiological function and/or support homeostasis that has been altered by somatic (body framework) dysfunction (ICD-10-CM Diagnosis Code M99.00-09), i.e. impaired or altered function of related components of the somatic system; skeletal, arthrodial and myofascial structures; and related vascular, lymphatic, and neural elements ^13^.

Osteopathic practitioners use their understanding of the relationship between structure and function to optimize the body’s self-regulating, self-healing capabilities. This holistic approach to patient care and healing is based on the concept that a human being is a dynamic functional unit, in which all parts are interrelated and which possesses its own self-regulatory and self-healing mechanisms. Two essential components of osteopathic health care are the structural evaluation of the patient for diagnosis and an array of manipulative techniques for treatment ^12^.

Aim of the structural examination is to locate somatic dysfunctions, while the array of manipulative techniques are used to relieve tissues tensions, improve blood flow and lymphatic drainage.

Although several studies document the effect of osteopathic manipulative treatment (OMT) in a population of pediatric patients ^14-16^, the medical literature lacks information about any potential benefits of the use of OMT in preterm infants. The only study published demonstrated a potential positive effect of OMT in reducing the likelihood of excessive LOS and gastro-intestinal symptoms ^17^.

Aim of this multicentre randomized controlled trial is to examine the association between OMT and LOS in a larger population.

METHODS AND ANALYSIS

Aim of the study

Primary outcome is to evaluate the effectiveness of OMT in reducing LOS in a sample of premature infants.

Secondary outcome of the study is to evaluate the difference in daily weight gain, number of episodes of vomit and regurgitation, stooling, use of enema, time to full enteral feeding (nipple and/or bottle) and NICU costs.

Study type

Interventional, multicentre nationwide single blinded randomized controlled trial. Preterm infants entering the trial will receive either routine medical care plus osteopathic evaluation and treatment or routine medical care plus osteopathic evaluation only (figure 1).

Participating NICUs

Patients will be recruited from three Italian secondary and tertiary NICUs at the public hospital in Pescara, Macerata and Monza.

The three participating NICUs are located throughout Italy in urban regions with catchment areas of about 40 000 to 150 000 inhabitants. Principal investigators of the participating NICUs are neonatologists and osteopaths with at least 5 years of osteopathic treatments in NICU and specialized neonatal osteopathic education.

Population

Participants who met the following inclusion criteria are considered eligible for the trial: male and female preterm infants entering the NICUs in the period between 1 July 2012 and 30 June 2013, preterms born in the same hospital of the referred NICU and free of medical complications and parents or legal guardian written informed consent.

Subjects presenting the following clinical conditions are excluded:

- gestational age <29 weeks
- gestational age >37 weeks
- first OMT performed after 14 days from birth
- genetic disorders
- congenital disorders
- cardiovascular abnormalities
- proven or suspected necrotized enterocolitis with or without gastrointestinal perforation
- proven or suspected abdominal obstruction
- pre/post surgery patients
- pneumoperitoneum
- atelectasis
- newborn from an HIV seropositive/drug addicted mother
- respiratory disorders
- transferred to/from other hospital
- admitted for preterminal comfort care (defined as neither intubation nor cardio-respiratory resuscitation)

Randomization process

All patients entering the study are sequentially allocated to the experimental and control arms using R (R core team, Vienna, Austria), an open source statistical software, as computer random number generator ^18^.

The type of randomization procedure is permuted-block (ratio 1:1).

The process of randomization is performed in the coordinating center.

Research investigators

Investigators will be grouped into three groups:

1. “consultant performing the randomization”
2. “osteopaths performing the evaluation”
3. “osteopaths performing the evaluation&treatment”

Consultant performing the randomization

An information technology consultant will be responsible for randomization prior to the arrival of the osteopaths to the NICU.

Osteopaths performing the evaluation

Osteopaths in this group will perform the osteopathic evaluation in all infants entering the trial, with no knowledge about patients allocation.

The osteopathic evaluation will be performed in the absence of the “osteopaths performing the evaluation&treatment”.

Osteopaths performing the evaluation&treatment

“Osteopaths performing the evaluation&treatment” will perform an osteopathic evaluation and treatment of preterm infants from group “A”.

Intervention provided into the trial

Patients from experimental and control groups will receive routine medical care.

After study enrollment, all patients are sampled in group A and group B.

1. Group A (OMT): patients under usual medical care plus osteopathic treatment.

Patients from this group will receive osteopathic care as follows: two treatments weekly for the entire length of stay in the unit. Patients from group A will also receive an osteopathic evaluation from the “osteopaths performing the evaluation”.

Osteopathic treatments will only be applied to patients from group A and will be performed only by the “osteopaths performing the evaluation&treatment”, not involved in the study design, data entry, statistical analysis.

Each OMT sessions involve the structural examination and specific manipulative procedures.

In newborns the structural exam is usually performed with the child lying down in the open crib or incubator. Diagnostic criteria for somatic disfunction are focused on tissue texture abnormalities, areas of asymmetry and misalignment of bony landmarks and the quality of motion, its balance and organization.

The second part of the OMT session is characterized by the use of a variety of therapeutic manual techniques, addressed to increase range of motion and resolve the somatic dysfunctions diagnosed.

Techniques to be used are in line with the benchmarks for osteopathic treatment available in the medical literature and are limited to myofascial release, balanced ligamentous/membranous tension, indirect fluidic and v-spread. The whole session will last 30 minutes, 10 minutes for evaluation and 20 minutes for treatment.

2. Group B (No OMT): control group.

Following the same schedule as group A, patients from group B will receive usual medical care and osteopathic evaluation only. The osteopathic evaluation will last 10 minutes. To maintain blinding of NICU personnel, the following 20 minutes osteopaths will keep their position close to the incubator or bed without touching the infant.

Osteopathic service will be provided twice a week, on Tuesdays and Fridays.

In case of critical preterm ’s health conditions (i.e. acute infections, per-acute emergency care) or supplemental neonatal medical screening during the osteopathic service, the aforementioned intervention can be temporally stopped for a given trial participant. The infant will not be either evaluated or treated. This is in line with hospital safety procedures and primary care priority intervention.

Allocation concealment and blinding

NICU staff are unaware of study design and outcomes.

NICU staff are blinded to patients allocation, since all infants will be touched by osteopaths from group A and B

Data entering and data export

Data collection will be performed using an ad hoc locally developed software called EBOM-GCCN.

EBOM-GCCN data set is an informatics tool that improves the efficiency and accuracy of data and has been developed to assist neonatologists, nurses and osteopaths in daily patients management.

The software consists of three sections:

- Section 1: Intended for use by neonatologists and nurses for recording patients' general details and all clinical information;

- Section 2: Intended for use by “osteopaths performing the evaluation&treatment”;

- Section 3: Intended for use by “osteopaths performing the evaluation”.

Records in section 1, 2 and 3 are exclusively and respectively accessible to NICU staff, “osteopaths performing the evaluation&treatment” and “osteopaths performing the evaluation”.

Nursing and medical records will be collected daily by the NICU staff, from the time the infants enters the unit to the time of discharge.

Osteopathic records will be collected twice weekly when the osteopathic service will be provided. “Osteopaths performing the evaluation&treatment” will collect data in relation to the structural examination and the techniques applied, while the “osteopaths performing the evaluation” will only collect data for the structural examination.

Data export will take place at the end of the study by the statistician from the coordinating center European Institute for Evidence Based Osteopathic Medicine (EBOM).

Measurements

To evaluate the effect of the treatment, standard measurements will be recorded. Data will be collected at the baseline (entry time, T0), every time the osteopathic service is provided and at the end of the stay in the unit (discharge time, T1). An expected average period of 4 weeks has been considered.

The following measurements will be included for the primary and secondary outcomes.

Primary outcome: length of stay (LOS)

LOS will be used as primary outcome and measured as the mean difference in days between T1 and T0.

According to international guidelines, the following physiological conditions are required for discharge: maintenance of body heat at room temperature, coordinated sucking, swallowing, and breathing while feeding; sustained pattern of weight gain; stability of cardiorespiratory function (no episodes of apnea/bradycardia for 2-5 days, free of supplemental oxygen support) ^8^.

Secondary outcomes

Secondary outcome measurements include the following parameters:

1. daily weight gain, referred as the net weight variation per day expressed in grams
2. episodes of vomit, the number of vomits per day
3. episodes of regurgitation, the number of regurgitations per day
4. episodes of stooling, the number of stools per day
5. use of enema, the number of enemas used per day
6. time to reach full enteral feeding, the number of days before autonomous feeding is achieved
7. NICU costs, calculated as NICU daily newborn expenses, according to local authorities, multiplied by the newborn’s LOS. Costs will be estimated in euros per day
8. side effects of treatment (osteopathy and clinical procedures)

In addition to these measurements, socio-demographic and clinical data will be collected and include:

1. newborn’s data: gender, gestational age, weight at birth and at entry, height, head circumference at birth, route, type and length of delivery, diagnosis at T0 and T1, associated pathologies
2. mother’s data: age, ethnicity, BMI, nationality, number of previous pregnancies, clinical medical condition during pregnancy, concurrent pathologies
3. father’s data: age, ethnicity, BMI, nationality, concurrent pathologies

Statistical analyses

All calculations will be performed at the coordinating center EBOM.

Statistical analyses will take into account the intention-to-treat analysis. Missing data will be handled using last observation carried forward (LOCF) imputation technique. Arithmetic means and standard deviation will be used for the general characteristics of the study population. Univariate statistical tests will be performed to compare the experimental group and control group at the baseline. A generalized linear model, linear regression, will be considered to study the independent effect of OMT on primary endpoint and secondary endpoints, taking into account all possible confounders. The significance level will be at α=0.01. Differences between the groups will be presented as mean with 95% CI or in categories with OR for categorical data.

The statistical program in use for randomization and data analyses is R ^18^.

Sample size

Sample size calculation used an effect size of 0.3 calculated from previous studies, considering a mean difference of 4 days between experimental and control group and a SD of 14. The statistical power is set at 0.90 and an alfa level equal to 0.01. This produces a sample size of 333 per group. To prevent loss of power, the sample size is increased up to 345 subjects per group. The whole sample (N=690) is then divided, with a final result of N=230 preterm infants for each NICU. A longer period of study enrollment has been considered a strategy to achieve the estimated sample size.

Coordinating center

The coordinating center EBOM consists of an executive division, President and Vice-President, an administration office, a group of researchers, an information technology consultant and a senior biostatistician.

The coordinating center collaborates with the A.I.O.T. - Accademia Italiana Osteopatia Tradizionale.

ETHICS AND DISSEMINATION

During the routine initial post-delivery consultation, the neonatologist approaches parents or legal guardians explaining the patient information sheet. Written informed consent will be then obtained from the above-mentioned guardians at study enrollment. Participation is voluntary and data collected will be sent to the coordinating centre using an anonymous identification for each patient.

The trail is approved by the ethical committee of Macerata hospital (n°22/int./CEI/27239) and it is under review by the other regional ethics committees. Moreover the trial has been registered on clinicaltrial.gov (identifier NCT01645137).

The expected disadvantages for the intervention provided in the trial are null as well as no potential side effects due to osteopathic care are predicted as shown by the recent osteopathic literature ^19^. However, any side effect will be recorded during the study period and appropriately discussed in the final paper.

Publication policy

The results of the trial will be published in peer-reviewed journals and presented at relevant congresses. The trial will be reported in accordance with the CONSORT recommendations.

DISCUSSION

The purpose of this multicentre single blinded RCT is to confirm the benefits of OMT in the care of preterm infants. In this trial, osteopathic care is not based on a predetermined protocol but on needs-based approach. Thus subjects can potentially be exposed to different techniques, leading to inter-subjects and intra-subjects OMT variability. Nevertheless the objective is to provide evidence of the impact of osteopathic approach rather than the association with a given manual protocol.

On the basis of quantitative data, the scope is to estimate the effect of OMT across different centers, proving generalizability on the effectiveness of osteopathy in NICUs. Well-designed researches are scarce in the osteopathic area, thus the present study will provide the most convincing evidence of relationship between exposure and effect.

To the best of our knowledge, this will be the first study in osteopathy applied to infants using gold standards methods that include a randomized multicentre approach, a single hub computer-based data collection and a large sample size stratified by risk factors. In addition to this, the statistics that will be used will be aimed to consider different sources of variability, to allow reliable results for the effectiveness of OMT. Finally, the expected benefits for the intervention provided in the trial are shorter period of hospitalization and improvements in the secondary outcomes, leading to enhanced quality of health service delivered and cost reductions.

Authors’ contribution

CF, PG and BG conceptualized, designed and wrote the protocol. RC, D’IC, FP, PFP and TL review the protocol for important intellectual content. All authors read and approved the final manuscript.

Financial statement: This research received no specific grant from any funding agency in the public, commercial or not-for-profit sectors.

Conflict of interest: Authors declare no conflict of interest.

Ackownledgments

Authors would like to thank Accorsi Alessandro, Cozzolino Vincenzo, D’Orazio Marianna, Gualdi Stefano, Lupacchini Mariacristina and Marinelli Benedetta for their precious contribution.

Bibliography

1. World Health Organization, March of Dimes, PMNCH, Save the Children. Born to soon: The Global Action Report on Preterm Birth. In: Eds CP Howson MK, JE Lawn, editor. Geneva: World Health Organization, 2012.

2. Owens PL TJ, Elixhauser A, Ryan K. Care of Children and Adolescents in U.S. Hospitals. In: 4 RHFBN, editor. *AHRQ Publication 04–0004*. Rockville, MD: Agency for Healthcare Research and Quality, 2003.

3. Clements KM, Barfield WD, Ayadi MF et al. Preterm birth-associated cost of early intervention services: an analysis by gestational age. *Pediatrics* 2007;119(4):e866-74.

1. Russell RB, Green NS, Steiner CA et al. Cost of hospitalization for preterm and low birth weight infants in the United States. *Pediatrics* 2007;120(1):e1-9.
2. Italian Ministry of Health. Rapporto annuale sull'attività di ricovero ospedaliero - dati SDO 2010. In. Roma: Ministero della salute; 2011.

6. Bakewell-Sachs S, Medoff-Cooper B, Escobar GJ et al. Infant functional status: the timing of physiologic maturation of premature infants. *Pediatrics* 2009;123(5):e878-86.

7. Eichenwald EC, Blackwell M, Lloyd JS et al. Inter-neonatal intensive care unit variation in discharge timing: influence of apnea and feeding management. *Pediatrics* 2001;108(4):928-33.

8. Hospital discharge of the high-risk neonate--proposed guidelines. American Academy of Pediatrics. Committee on Fetus and Newborn. *Pediatrics* 1998;102(2 Pt 1):411-7.

9. Mezzacappa MA, Rosa AC. Clinical predictors of abnormal esophageal pH monitoring in preterm infants. *Arq Gastroenterol* 2008;45(3):234-8.

10. Mihatsch WA, von Schoenaich P, Fahnenstich H et al. The significance of gastric residuals in the early enteral feeding advancement of extremely low birth weight infants. *Pediatrics* 2002;109(3):457-9.

11. Duman N, Utkutan S, Ozkan H et al. Are the stool characteristics of preterm infants affected by infant formulas? *Turk J Pediatr* 2000;42(2):138-44.

12. World Osteopathic Health Organization. WOHO. Osteopathic glossary, 2011.

13. American Association of Colleges of Osteopathic Medicine. Glossary of Osteopathic Terminology, 2002.

14. Hayden C, Mullinger B. A preliminary assessment of the impact of cranial osteopathy for the relief of infantile colic. *Complement Ther Clin Pract* 2006;12(2):83-90.

15. Vandenplas Y, Denayer E, Vandenbossche T et al. Osteopathy may decrease obstructive apnea in infants: a pilot study. *Osteopath Med Prim Care* 2008;2:8.

16. Philippi H, Faldum A, Schleupen A et al. Infantile postural asymmetry and osteopathic treatment: a randomized therapeutic trial. *Dev Med Child Neurol* 2006;48(1):5-9; discussion 4.

17. Pizzolorusso G, Turi P, Barlafante G et al. Effect of osteopathic manipulative treatment on gastrointestinal function and length of stay of preterm infants: an exploratory study. *Chiropractic & Manual Therapies* 2011;19(15).

18. R Development Core Team. R: A language and environment for statistical computing. *R Foundation for Statistical Computing*. Vienna, Austria., 2010.

19. Hayes NM, Bezilla TA. Incidence of iatrogenesis associated with osteopathic manipulative treatment of pediatric patients. *J Am Osteopath Assoc* 2006;106(10):605-8.


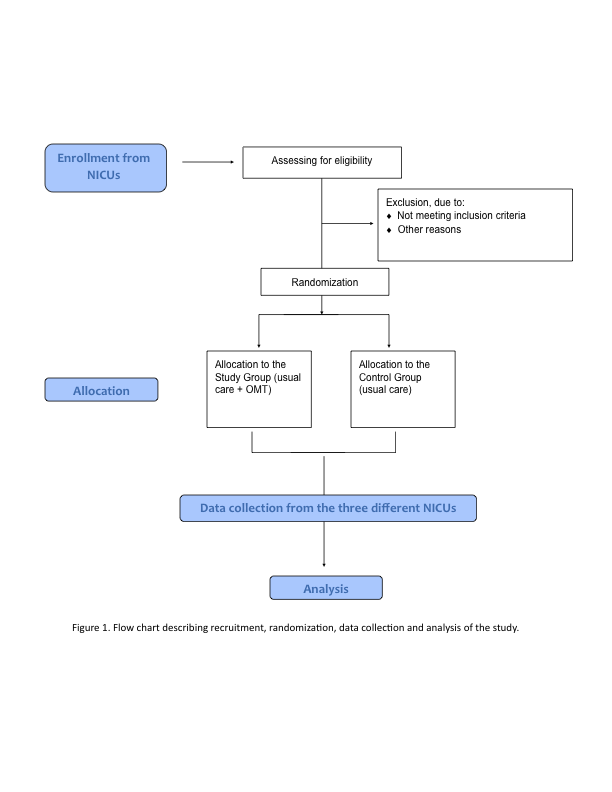

Supplement: S1 Protocol — Description of the research protocol used for the current trial. (DOC) [file pone.0127370.s002.doc]
